# Supplementary material for: Biphasic regulation of chondrocytes by Rela through induction of anti-apoptotic and catabolic target genes
Source: Nat Commun. 2016 Nov 10;7:13336. doi: 10.1038/ncomms13336 (PMC5109547; doi:10.1038/ncomms13336)
Supplement: Supplementary Information — Supplementary Figures 1-7 and Supplementary Tables 1-3 [file ncomms13336-s1.pdf]

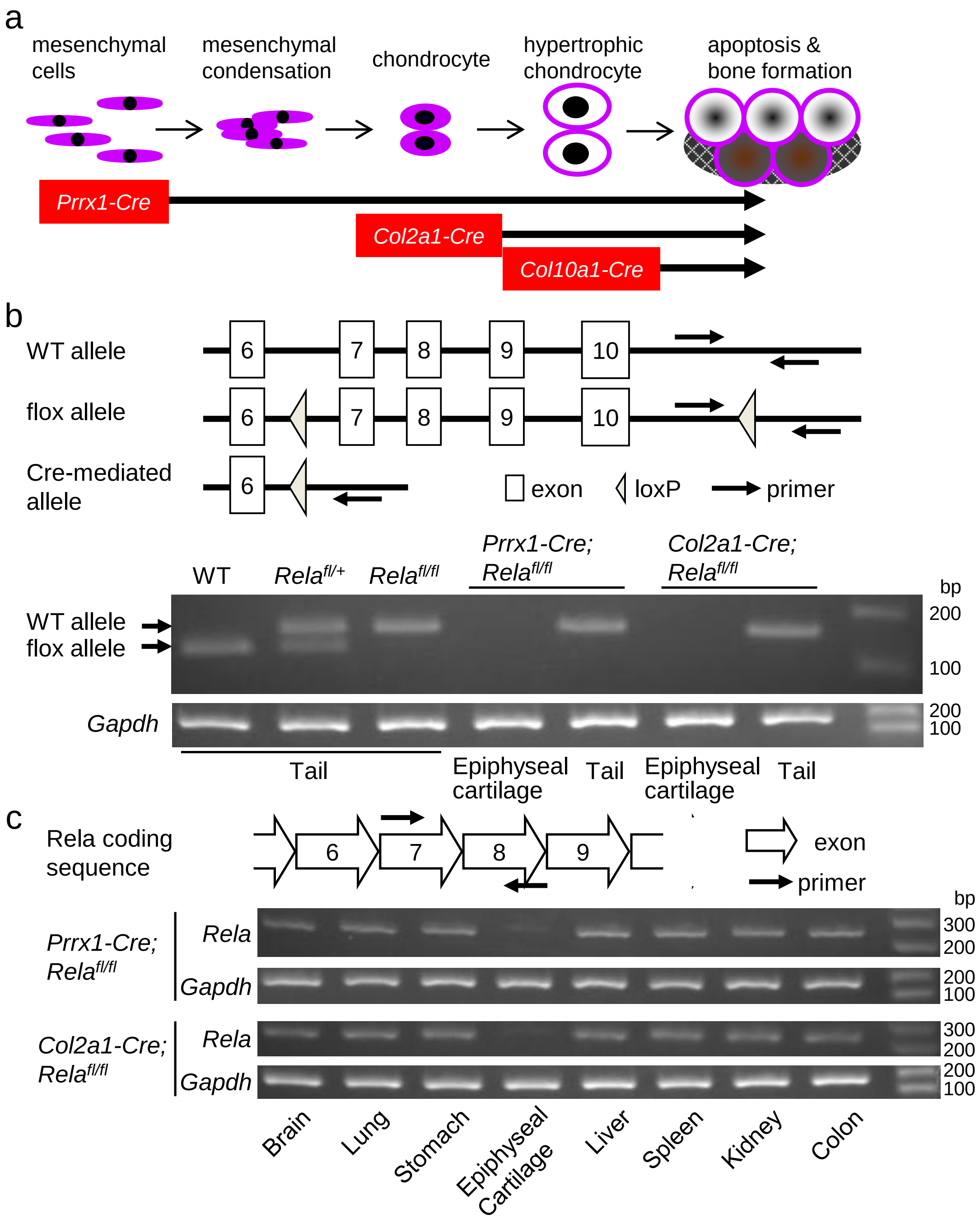

Supplementary Figure 1

Cre mice used for *in vivo* analyses of Rela during skeletal development. (**a**) Scheme of Cre expression in the three mouse strains during chondrocyte generation and endochondral ossification. (**b**) Genotyping of *Rela-flox* mice. A primer set for genotyping was designed to detect the latter loxP site. WT or floxed alleles were not detected in genomic samples obtained from epiphyseal cartilage of *Prrx1-Cre;Rela*$^{fl/fl}$ and *Col2a1-Cre;Rela*$^{fl/fl}$ mice, indicating tissue-specific recombination in both mouse lines. (**c**) RT-PCR analysis of *Rela* using mRNA samples obtained from various tissues of *Prrx1-Cre;Rela*$^{fl/fl}$ and *Col2a1-Cre;Rela*$^{fl/fl}$ mice, indicating tissue-specific knockdown of *Rela* in both mouse lines.

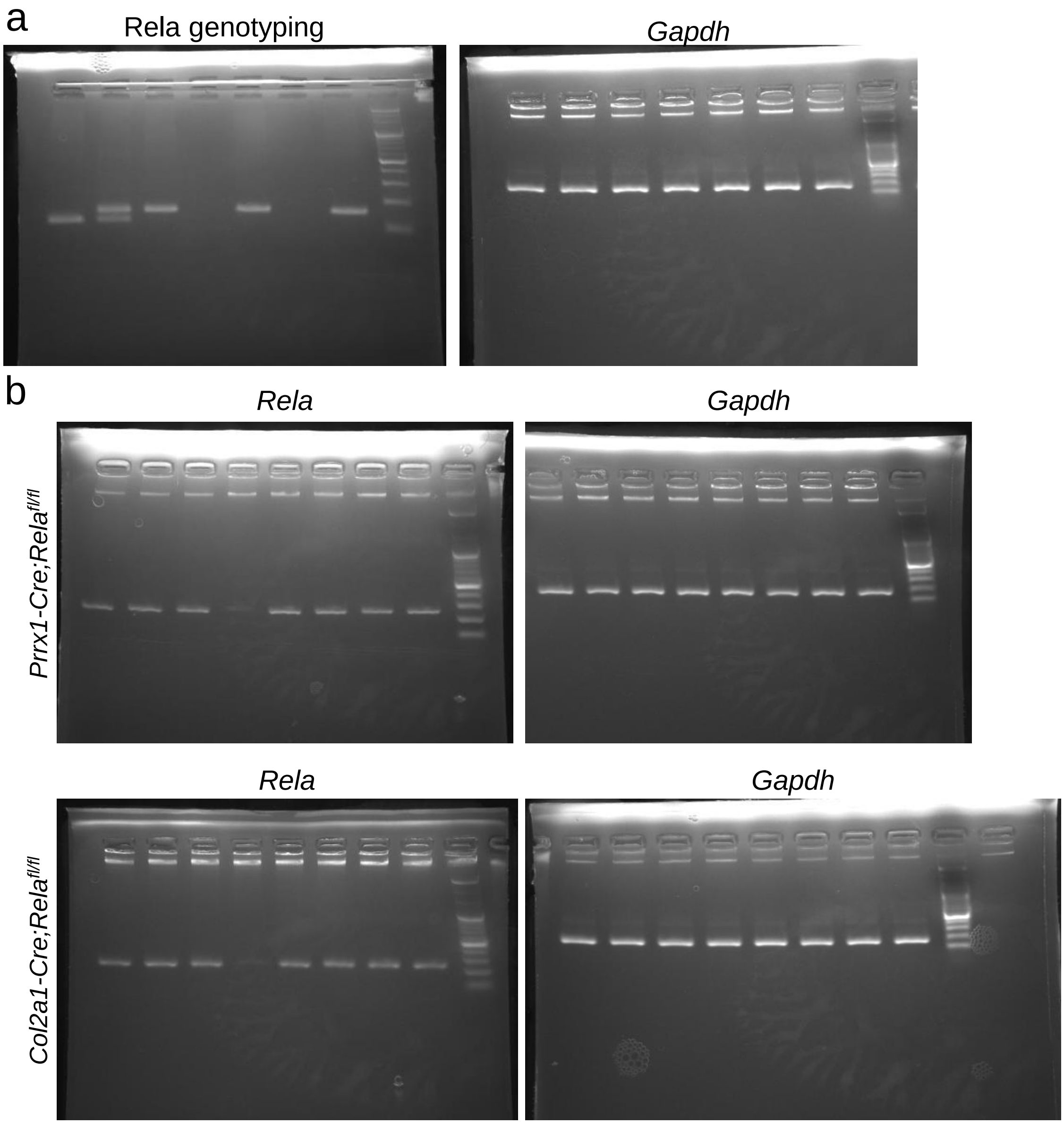

Supplementary Figure 2
Original images of the agarose gel electrophoresis shown in Supplementary Fig.1b, and c.
(**a**) Original images of Supplementary Fig.1b. (**b**) Original images of Supplementary Fig.1c.

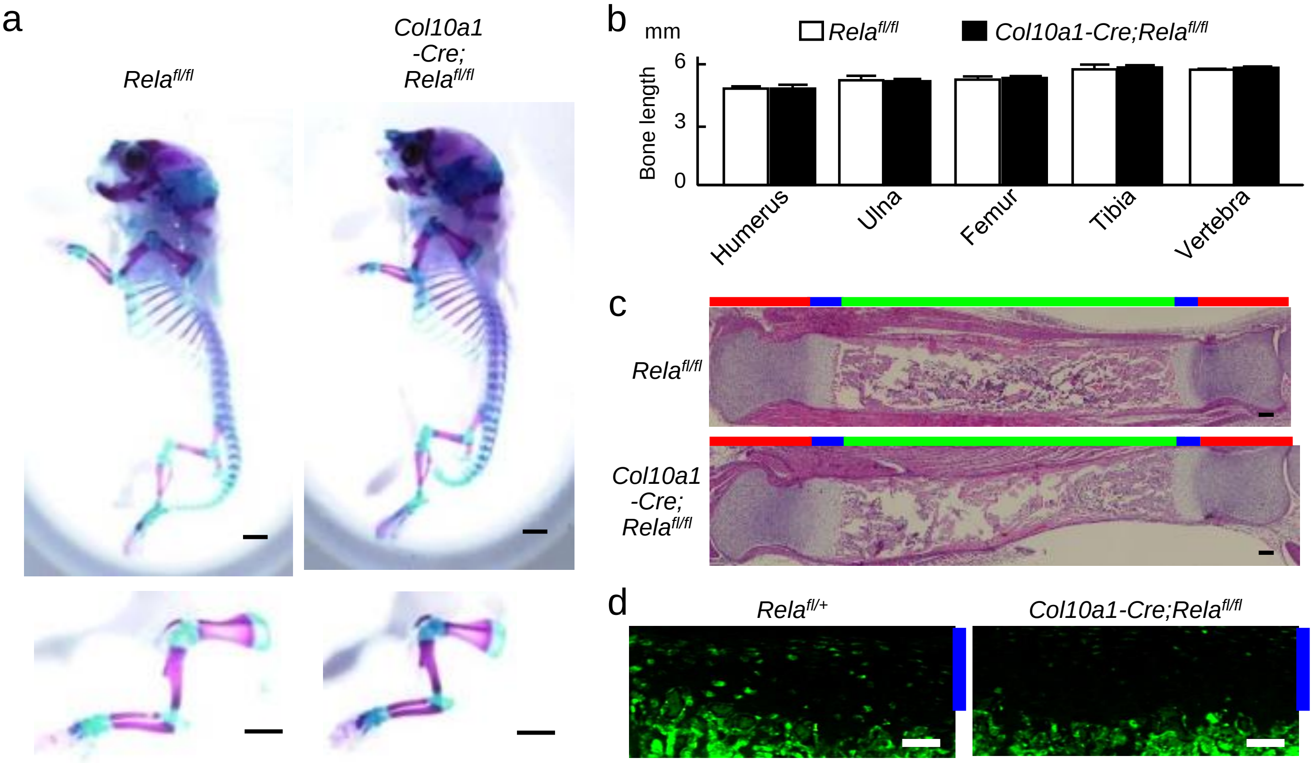

Supplementary Figure 3

Skeletal development of *Col10a1-Cre;Rela$^{fl/fl}$* mice. (**a**) Double staining with Alizarin red and Alcian blue of the whole skeleton (left) and upper extremities (right) of *Rela$^{fl/fl}$* and *Col10a1-Cre;Rela$^{fl/fl}$* littermate embryos (E18.5). Scale bars, 1 mm. (**b**) Length of long bones and vertebrae (first to fifth lumbar spines) of *Rela$^{fl/fl}$* and *Col10a1-Cre;Rela$^{fl/fl}$* littermate embryos (E18.5). Data are expressed as the means ± s.d. of five mice per group. (**c**) H&E staining of whole tibias of *Rela$^{fl/fl}$* and *Col10a1-Cre;Rela$^{fl/fl}$* littermate embryos (E18.5). Scale bars, 100 μm. Upper bars indicate lengths of the proliferative zone (red), hypertrophic zone (blue), and bone area (green). (**d**) Immunofluorescence of Rela in the proximal tibia of *Rela$^{fl/fl}$* and *Col10a1-Cre;Rela$^{fl/fl}$* littermate embryos (E18.5). Right blue bars indicate the hypertrophic zone. Scale bars, 50 μm.

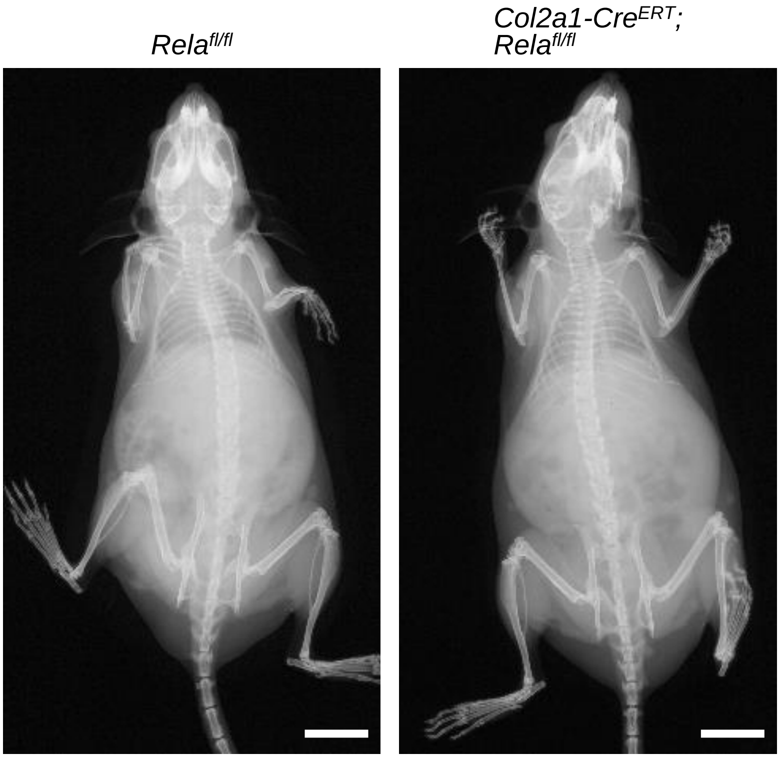

Supplementary Figure 4

Skeletal development of *Col2a1-Cre*^{ERT};*Rela*$^{fl/fl}$ mice. Plain radiophotograph of *Rela*$^{fl/fl}$ and *Col2a1-Cre*^{ERT};*Rela*$^{fl/fl}$ littermates (7 weeks old, without tamoxifen induction). Scale bars, 10 mm.

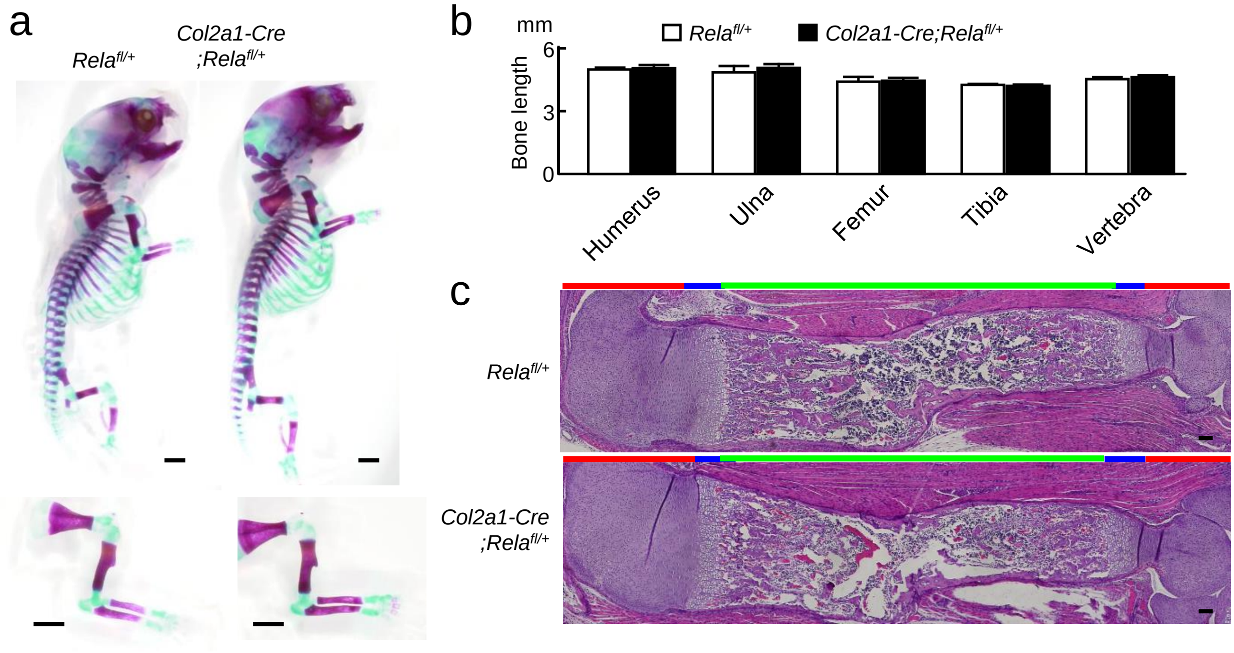

Supplementary Figure 5

Skeletal development of *Col2a1-Cre;Rela*$^{fl/+}$ mice. (**a**) Double staining with Alizarin red and Alcian blue of the whole skeleton (top) and upper extremities (bottom) of *Rela*$^{fl/+}$ and *Col2a1-Cre;Rela*$^{fl/+}$ littermate embryos (E18.5). Scale bars, 1 mm. (**b**) Length of long bones and vertebrae (first to fifth lumbar spines) of *Rela*$^{fl/+}$ and *Col2a1-Cre;Rela*$^{fl/+}$ littermate embryos (E18.5). Data are expressed as the means ± s.d. of three *Rela*$^{fl/+}$ mice and five *Col2a1-Cre;Rela*$^{fl/+}$ mice. (**c**) H&E staining of whole tibias of *Rela*$^{fl/+}$ and *Col2a1-Cre;Rela*$^{fl/+}$ littermate embryos (E18.5). Scale bars, 100 µm. Upper bars indicate lengths of the proliferative zone (red), hypertrophic zone (blue), and bone area (green).

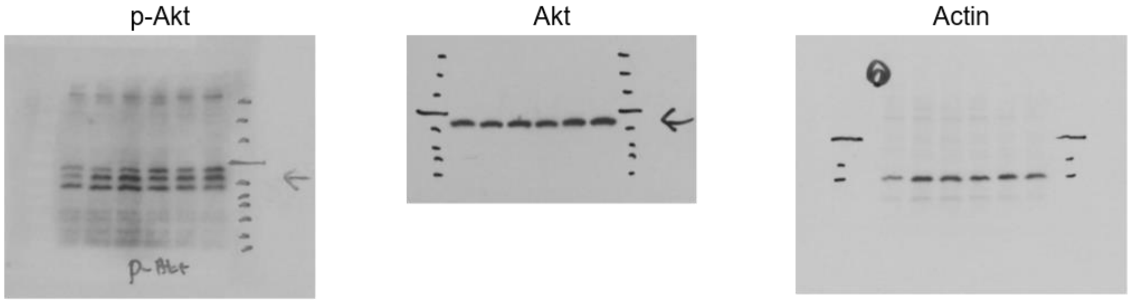

Supplementary Figure 6
Original images of the immunoblots shown in Fig. 5d.

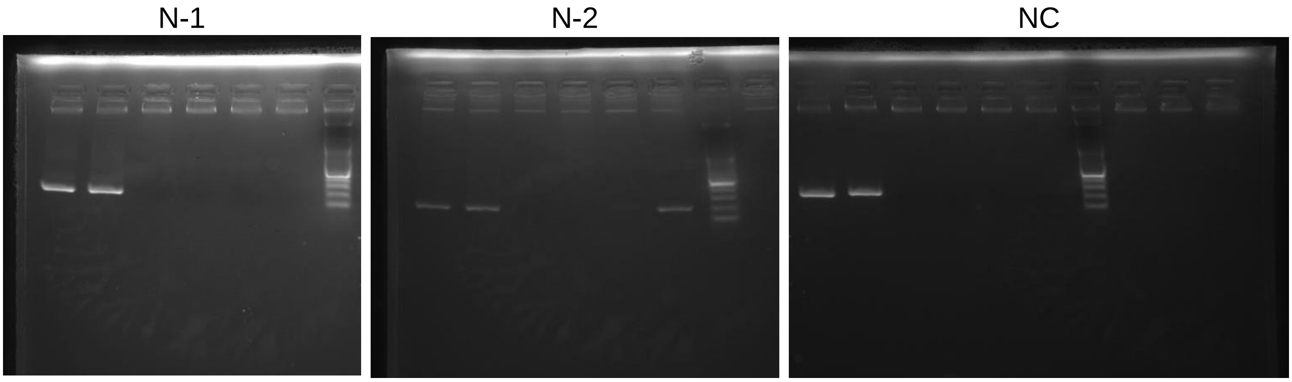

Supplementary Figure 7
Original images of the agarose gel electrophoresis of ChIP-PCR shown in Fig. 6e.

Supplementary Table 1. Primers used for genotyping.

Rela	F	gaagctggtctgccaagaac
	R	catcaaagtcagccaggtca
Cre	F	gcggtctggcagtaaaaactatc
	R	gtgaaacagcattgctgtcactt
Gapdh	F	tgcaccaccaactgcttagc
	R	acccaaaagggacacctaca

Supplementary Table 2. Primers used for real-time RT-PCR.

Rela	F	aggtgtatttcacgggacca
	R	cataggtccttttgcgcttc
Col2a1	F	gccaagacctgaaactctgc
	R	gccatagctgaagtggaagc
Acan	F	ccaaaccagcctgacaactt
	R	tctagcatgctccaccactg
Sox9	F	cgactacgctgaccatcaga
	R	agactggttgttcccagtgc
Cdkn1c	F	aggagcaggacgagaatcaa
	R	acgtttggagagggacacc
Ccnd1	F	agtgcgtgcagaaggagatt
	R	cacaacttctcggcagtcaa
Traf2	F	gccagattttggagcagaag
	R	ccagctgttgcaccttgtta
Cflar	F	acagagtgaggcggtttgac
	R	ccttggctatcttgcctctg
Birc2	F	gctcagaatcaaaggccaag
	R	caccaggctcctactgaagc
Pik3ca	F	actgttcagagaggccagga
	R	cggttgcctactggttcaat
Birc3	F	ggggacagtcctgttgaaaa
	R	ggctagagcacaggttggag
Map3k14	F	caaatcctctcctgcctcag
	R	tgttcgccattgagagactg
Pik3r1	F	agccgccagctctgataata
	R	tctccccagtaccattcagc
Ikbkg	F	ggtggagagactgagcttgg
	R	ctaaagcttgccgatccttg
Ppp3cc	F	gaaccaagcaactggctttc
	R	tccaacaaaaggcaaagacc
Adamts5	F	gctactgcacagggaagagg
	R	tgcatatttgggaacccatt
Hif2a	F	tgagttggctcatgagttgc
	R	ctcacggatctcctcatggt
Actb	F	agatgtggatcagcaagcag
	R	gcgcaagttaggttttgtca
Gapdh	F	tgcaccaccaactgcttagc
	R	ggatgcagggatgatgttct

Supplementary Table 3. Primers used for ChIP-PCR

N-1	F	ctccggttcacccttttcac
	R	accccaggctttttcgag
N-2	F	ggacgtgtagagccagagca
	R	gagcccacaccaaccaga
NC	F	aggctagctgtcggagacg
	R	tgccagtgacaggaagagag
